# Attachment insecurities, continuing bonds, and grief among family caregivers of terminally ill cancer patients: A longitudinal study

**DOI:** 10.1017/S1478951525100412

**Published:** 2025-07-18

**Authors:** Wan-Lin Lee, Yaw-Sheng Lin, Emily T. Liu, Chih-Tao Cheng, Shu Kao

**Affiliations:** 1 Department of Clinical Psychology, Fu Jen Catholic University, New Taipei City, Taiwan; 2 Department of Psychology, National Taiwan University, Taipei City, Taiwan; 3 Department of Psychiatry, Koo Foundation Sun Yat-Sen Cancer Center, Taipei City, Taiwan; 4 Department of Psychology and Social Work, National Defense University, Taipei City, Taiwan; 5 Department of Family Medicine, Taipei Veterans General Hospital, Taipei City, Taiwan

**Keywords:** Pre-loss grief, attachment insecurities, family caregivers, continuing bonds, Longitudinal study

## Abstract

**Objectives:**

The present study investigated the associations among pre-loss grief, relational closeness, attachment insecurities, continuing bonds (CBs) with the deceased person, and the post-loss adjustment of the caregivers of patients with terminal cancer.

**Methods:**

Data were collected in the hospice department of a cancer center in northern Taiwan; 66 bereaved caregivers completed both pre-loss and post-loss scales. The measures used for the pre-loss phase included the Hogan Grief Reaction Checklist (HGRC; pre-loss version), the Experiences in Close Relationship – Relationship Structures Questionnaire (ECR-RS), and the Inclusion of Other in the Self Scale. The measures used 6–12 months after the death of the patients were the HGRC (post-loss version) and the Continuing Bond Scale (CBS).

**Results:**

Pre-loss grief and externalized CBs had a significant impact on the amount of post-loss grief, indicating that pre-loss grief and ongoing transformation of relationships after patients’ death may be predictors of caregivers’ post-loss grieving.

**Significance of results:**

This longitudinal study provides preliminary evidence that pre-loss grief and the relationship with the patient are key to caregivers’ post-loss adjustment, suggesting that psychosocial intervention focuses on caregivers’ pre-loss grief and relationship quality with the patient during palliative care.

## Introduction

During the end-of-life care phase, family caregivers often suffer from their inner sadness resulting from the impending death. Grief severity in family caregivers before the death of a loved one is referred to as pre-loss grief (Lindauer and Harvath 2014; Nielsen et al. 2017; Singer et al. 2022) or pre-death grief (Holm et al. 2019). A recent systematic review (Treml et al. 2021) concluded that caregivers with high levels of pre-loss grief and low levels of preparedness for impending death were associated with poor bereavement adjustment. While the underlying mechanism remains unclear, it is essential to examine possible psychological factors or processes to explain the relationship between pre-loss grief and post-loss adjustment.

The experience of caring for terminally ill cancer patients may involve both challenges and personal growth (Tang 2019). Therefore, assessing the positive aspects of care can help caregivers identify the meaning of the experience and better adjust throughout palliative care. A systematic review showed that positive aspects of caregiving include an enhanced relationship with the patient, feeling rewarded, a sense of personal growth, and a perception of personal satisfaction (Li and Loke 2013). A recent study also revealed that identifying meaning in the caregiving experience could be a protective factor favoring adaptation (Palacio and Limonero 2020). These studies support a potential relationship between the caring experience, the caregiver–patient relationship, and the personal growth of caregivers.

Caregivers’ relationships with terminally ill patients play a crucial role in their subsequent bereavement adjustment. Attachment, the sense of psychological security in an individual’s relationships with others, is fundamental for developing healthy interpersonal relationships (Bowlby 1969, 1973, 1980, 1982; Wayment and Vierthaler 2002). In adult attachment research, attachment insecurities have been conceptualized in terms of 2 dimensions: anxiety and avoidance (Cohen and Katz 2015; Fraley et al. 2011; Tsilika et al. 2015). Evidence indicates that attachment insecurities can affect patients’ and caregivers’ caring quality and well-being (e.g., Nicholls et al. 2014; Tsilika et al. 2015). One recent study (Sękowski et al. 2024) showed that attachment anxiety has a positive relationship with posttraumatic growth via intrusive and deliberate rumination. Research has also explored the possibility of insecure attachment style being a potential risk factor for caregivers to develop prolonged grief disorder (Liljeroos et al. 2024). While a meta-analysis concluded that longitudinal analyses showed neither anxious nor avoidant attachment styles increase prolonged grief symptoms (Eisma et al. 2023), researchers suggested that disorganized attachment style might moderate the effect between avoidant attachment style and prolonged grief symptom severity (Sekowski and Prigerson 2022b). In sum, caregivers’ attachment insecurities may affect their caring experience, likely extending to their bereavement adjustment.

During the last decade, continuing bonds (CBs), which highlights the importance of continuing relationships with the deceased person during bereavement adjustment, has initiated a revolutionary change in grief theory (Klass et al. 1996). Field et al. (2005) proposed an attachment-based CB theory to explain how attachment might interact with the formation of CBs. Individuals with insecure attachment styles tend to establish maladaptive CBs with the deceased person. To elaborate on the theory, Field and Filanosky (2009) distinguished CBs into 2 types: internalized and externalized. Internalized CBs, which function as a safe and stable inner resource, result from successful internalization of the deceased. In contrast, externalized CBs are expressed as illusions and hallucinations regarding the physical presence of the deceased person and are a maladaptive means of coping with grief.

To the best of our knowledge, studies investigating the longitudinal effects of pre-loss grief, pre-loss growth, and attachment insecurities on caregivers’ post-loss adjustment have been limited. Prior studies focused primarily on the association between pre-loss grief and prolonged grief symptomatology, and aimed to identify the potential risk factors for prolonged grief disorder in the pre-loss phase (Nielsen et al. 2017; Stroebe et al. 2010; Zordan et al. 2019). Limited research has focused on the association between normal pre-loss grief and post-loss grief (Holm et al. 2019). Moreover, empirical findings on the association between attachment insecurities and types of CBs have been inconsistent (Ho et al. 2013; Root and Exline 2014; Yu et al. 2016). One reason for this inconsistency may involve the cross-sectional nature of most CB studies (Root and Exline 2014; Yu et al. 2016). Given such, a longitudinal study is needed to elucidate how caregivers’ attachment insecurities with a patient in the caregiving phase may affect caregivers’ grief and CB expressions when they face the death of patients.

Previous studies suggested that relational closeness might be a risk factor for prolonged grief disorder (Harrison et al. 2022; Sekowski and Prigerson 2022). However, Taiwanese society values relational harmony over individual fulfillment (Kim et al. 2006). Thus, caregivers may benefit more from close relationships during the caring phase than caregivers from non-Confucian cultures. Generally speaking, caring experiences can improve relational closeness between the caregiver and the patient by resolving unfinished business or previous conflicts. Considering this, the present study also measured the relational closeness between the caregiver and the patient during the palliative care phase.

The present study explored the longitudinal effects of pre-loss grief, pre-loss growth, relational closeness, and attachment insecurities on caregivers’ post-loss adjustment. We also assessed caregivers’ CB expressions 6–12 months after the patient’s death with the aim of exploring the association between pre-loss attachment insecurities and post-loss CB expressions. The present study sought to answer the following research questions: (1) What are the longitudinal effects of pre-loss grief, pre-loss growth, relational closeness, and attachment insecurities on post-loss grief and post-loss growth? (2) Are attachment insecurities more associated with externalized CBs, as predicted by CBs theory?

This study measures pre- and post-loss grief to assess the severity of grief rather than prolonged grief symptoms (Prigerson et al. 2009; Sękowski et al. 2024). Research has shown a strong correlation between grief severity and prolonged grief symptoms (Nielsen et al. 2017). Given such, we suggest that lower grief severity might indicate better adjustment to bereavement.

## Methods

### Participants

A total of 66 bereaved caregivers (43 females and 23 males) participated in the study. Data collection took place mainly between January 2017 and December 2019 (*n* = 59), with several additional data collected between July and October 2020 (*n* = 7). The age of the participants ranged from 18 to 69 years, with a mean age of 46.82 years (*SD* = 11.67). The majority of the sample was well-educated, and most of the patients being cared for were the parents or spouses of the participants (see Table 1 for the summary of the demographic information).

**Table 1.** Demographic information of participants (*N* = 66)

	Mean ± SD/*n* (%)
Gender	
Female	43 (65.2)
Male	23 (34.8%)
Age	47 ± 11
Relationship (to the participant)	
Parent	41 (62.1)
Spouse	19 (28.8)
Sibling	6 (9.1)
Education	
Primary school	1 (1.5)
Middle school	1 (1.5)
Senior high school	10 (15.2)
Vocational school	10 (15.2)
University	33 (50.0)
Graduate school	11 (16.7)

### Measures

#### Hogan Grief Reaction Checklist

The Hogan Grief Reaction Checklist (HGRC), designed to measure the multiple dimensions of the bereavement process, is a 61-item questionnaire rated on a self-reported 5-point Likert scale (Hogan and Schmidt 2015). In our previous study (Lee et al., 2024), we had obtained permission from Dr. Hogan to translate the HGRC into the Traditional Chinese Version (TC-HGRC). The TC-HGRC comprises 6 factors corresponding to the items in the original version of the HGRC. The personal growth subscale is scored independently to represent respondents’ growth after the experience of loss. The total grief score is calculated by summing 5 subscales, excluding the personal growth subscale. The test–retest reliability of the TC-HGRC subscales after 1 month were acceptable, (Person’s correlation coefficients: despair, .70; panic behavior, .73; personal growth, .56; blame and anger, .77; detachment, .83; and disorganization, .82, Author et al., 2022). In the present study, the internal consistencies (Cronbach’s alphas) for the 6 factors in the TC-HGRC are as follows: For the pre-loss version: despair, .84; panic behavior, .90; blame and anger, .77; detachment, .80; and disorganization, .83; personal growth, .70. For the total grief score: Cronbach’s *α* = .95; For the post-loss version: despair, .93; panic behavior, .92; blame and anger, .80; detachment, .89; and disorganization, .88; personal growth, .87. For the total grief score: Cronbach’s *α* = .97.

To assess the pre-loss state of the caregivers, we slightly modified the wording of 2 items in the TC-HGRC to indicate that the patients were still alive. Caregivers’ pre-loss growth was measured by the subscale of personal growth, which included the following items: “I have learned to cope better with life,” “I feel as though I am a better person,” “I have a better outlook on life,” and “I have more compassion for others.” The scale was used to assess participants’ pre-loss grief (pre-loss phase) and post-loss grief (post-loss phase).

#### The Experiences in Close Relationships–Relationship Structures Questionnaire

The Experiences in Close Relationships–Relationship Structures Questionnaire (ECR-RS) is a 7-point self-report scale assessing attachment insecurities (i.e., attachment-related avoidance and anxiety) toward significant attachment figures in young adulthood (Fraley et al. 2011). The scale comprises 9 items: 6 items measure avoidance, and 3 items measure anxiety. The Traditional Chinese version of the ECR-RS has been found to have satisfactory internal reliabilities (Cronbach’s *α* = .86–.90 for avoidance; Cronbach’s *α* = .90 – .91 for anxiety) and test–retest reliabilities (*r* = .73 for avoidance; *r* = .70 for anxiety) (Lin 2016). In the present study, Cronbach’s *α* = .82 for avoidance; Cronbach’s *α* = .66 for anxiety. The scale was used in the pre-loss phase.

#### The Inclusion of Other in the Self Scale

The Inclusion of Other in the Self scale (IOS) (Aron et al. 1992) was used to assess caregivers’ relational closeness with the patients during the pre-loss phase. As a diagram-like measure, the scale contains 7 pairs of overlapping circles, with each pair overlapping slightly more than the preceding pair. Respondents should select 1 out of 7 Venn-like diagrams that best depict their relational closeness with another person. In the present study, we adapted IOS to ask caregivers which best depicts their relationship with the patient during palliative care. The IOS has been shown to possess good test–retest reliability and convergent and discriminant validity (Aron et al. 1992). The Chinese version also has acceptable test-retest reliability (*r* = 0.739, *p* < 0.01; Ke 2021).

#### The Continuing Bond Scale

The original Continuing Bond Scale (CBS) (Field and Filanosky 2009) is a 16-item measure used to identify the ongoing relationships with the deceased patient, and was used in the post-loss phase. The CBS comprises 2 subscales: internalized and externalized CB. In the present study, we adopted the Traditional Chinese version developed by Ho et al. (2013). Ho et al. (2013) reported that the Cronbach’s alpha of the Traditional Chinese version of the Continuing Bond Scale (C-CBS) was .92, and that the Cronbach’s alphas of the internalized and externalized CB subscales were .92 and .84, respectively. In the present study, Cronbach’s alphas of the internalized and externalized CB subscales were .89 and .79, respectively.

### Procedure and ethical considerations

Participants were recruited through referrals from a palliative care team in the cancer center of a hospital. Informed consent was obtained in the end-of-life care phase. The inclusion criteria were as follows: Caretakers who were at least 18 years of age, with no psychiatric diagnosis during the caring phase. The duration of hospice care for the patients cared for ranged from days to months before the patients died, with a median duration of approximately 2 weeks. If there was more than 1 caregiver met the inclusion criteria in a family, only the primary caregiver would be invited to participate in the research. However, the whole family in the palliative and hospice department would be approached and cared for by psychologists during the first few days of the patients’ admission, whether they decided to participate in the current research or not. Individuals who agreed to participate in the study were asked to complete the demographic information and the TC-HGRC (pre-loss version), ECR-RS, and the IOS.

After the patient passed away at least 6 months, psychologists started to contact the caregivers by telephone to provide them with psychosocial support and psychological information on bereavement care. This phone call was conducted between 6 and 12 months after the patient’s death. The psychologists then confirmed with the caregivers that they wished to participate in the follow-up study while they were grieving. They were asked to complete the TC-HGRC and TC-CBS. Among the 96 caregivers, 10 participants declined to participate the follow-up studies, and 20 participants did not respond. The overall response rate was 68.8%.

The Institutional Review Board approval had been obtained for the present study (KFSYSCC-IRB: 20180504A). Moreover, the present study was a part of a multiyear research project sponsored by the Ministry of Science and Technology in Taiwan. The entire project had received approval from a cancer center institutional ethics review board in 2015 (protocol number KFSYSCC-IRB-20150402A).

## Results

We used IBM SPSS 25 to conduct the following analyses. First, we conducted correlation and differential analyses to examine the impact of sociodemographic variables (age, gender, educational levels, and relationship types) on caregivers’ post-loss grief and growth. Given that only 1 respondent (*n* = 1) has a certain level of education, education levels have been transformed into a binary variable, higher education versus less than university education, in the following analyses. The results indicate that there was a marginal but not significant negative correlation between age and post-loss grief. (*r* = − .23, *p* = .061), while none of the sociodemographic variables significantly correlated or differed in post-loss grief scores. For personal growth, only gender had a marginal but not significant effect on HGRC-personal growth subscale; that is, women had slightly higher scores on the HGRC-personal growth subscale (M = 51.57, SD = 21.14, *F* (1, 64) = 3.31, *p* = .074) in comparison to men (M = 43.13, SD = 9.25).

Then, bivariate correlation analyses were conducted to examine potential correlations among the caregivers’ pre-loss and post-loss variables. Table 2 presents the descriptive statistics and bivariate correlations between the study variables.

**Table 2.** Descriptive statistics and bivariate correlations between study variables

	M	SD	1	2	3	4	5	6	7	8	9
1. Relational closeness	4.56	1.93	1	.33**	−.35**	−.04	−.05	−.10	.00	.26*	−.14
2. Pre-loss growth	41.7	10.37		1	−.18	−.08	−.11	.10	.04	.44**	−.07
3. Pre-loss grief	95.34	25.91			1	.04	.17	.24	.12	−.29*	.35**
4. Attachment avoidance	3.13	1.44				1	.33**	−.18	−.17	−.14	−.2
5. Attachment anxiety	2.3	1.09					1	−.23	−.02	−.12	.00
6. Internalized CB	3.23	0.83						1	.58**	−.05	.18
7. Externalized CB	1.12	0.43							1	.05	.37**
8. Post-loss growth	48.63	18.28								1	.05
9. Post-loss grief	101.55	29.71									1

* *p* < 0.05.

** *p* < 0.01.

Relational closeness: Inclusion of Other in the Self Scale; Pre-loss growth: HGRC-personal growth subscale during end-of-life care; Pre-loss grief: pre-loss version of HGRC; Internalized CB: CBS internalized subscale; Externalized CB: CBS externalized subscale; growth-post: HGRC personal growth subscale after loss; post-loss grief: HGRC scores after loss.

As shown in Table 2, relational closeness during end-of-life care (IOS) was positively correlated with both pre-loss growth (*r* = .33, *p* = .007) and post-loss growth (*r* = .26, *p* = .037). IOS was negatively correlated with pre-loss grief (*r* = − .35, *p* = .004), which was in line with our research hypothesis. Not surprisingly, pre-loss grief was positively correlated with post-loss grief (*r* = .35, *p* = .004), and externalized CB was also positively correlated with post-loss grief (*r* = .37, *p* = .002). Contrary to our prediction, neither attachment anxiety nor attachment avoidance had a significant correlation with both pre-loss and post-loss grief. For the relationship between attachment insecurities and CB expression, both attachment avoidance and attachment anxiety were not significantly correlated with externalized CBs. Attachment anxiety was negatively correlated with internalized CBs, although this correlation was only marginally significant (*r* = − .23, *p* = .077).

We conducted partial correlation analyses to control for the effects of gender and age. This allowed us to examine the possible impact of pre-loss grief, pre-loss growth, attachment insecurities, relational closeness, and CB expressions on post-loss grief and post-loss growth. Results are shown in Table 3. Not surprisingly, pre-loss grief was positively correlated with post-loss grief (*r* = .30, *p* = .015). Attachment avoidance (*r* = − .25, *p* = .045), but not attachment anxiety (*r* = − .03, *p* = .82), was negatively correlated with post-loss grief. Externalized CBs (*r* = .39, *p* < .001) but not internalized CBs (*r*= .20, *p* = .119) strongly correlated with post-loss grief. There was a significant positive correlation between both pre-loss growth (*r* = .46, *p* < .001) and relational closeness during the caring phase (*r* = .27, *p* = .035) with post-loss growth. Additionally, pre-loss grief negatively correlated with post-loss growth (*r* = − .28, *p* = .024).

**Table 3.** Partial correlations between study variables after controlling for demographic variables

	1	2	3	4	5	6	7	8	9
1. Pre-loss growth	1	−.14	−.06	−.09	.32*	.10	.04	.46***	−.02
2. Pre-loss grief		1	.00	.15	−.34*	.25*	.13	−.28*	.30*
3. Attachment avoidance			1	.32*	−.02	−.17	−.16	−.15	−.25*
4. Attachment anxiety				1	.04	−.22	−.01	−.11	−.03
5. Relational closeness					1	−.11	−.00	.27*	−.12
6. Internalized CB						1	.58***	−.03	.20
7. Externalized CB							1	.07	.39***
8. Post-loss growth								1	.08
9. Post-loss grief									1

* *p* < .05.

*** *p* < .001.

Partial correlation control variables: gender and age.

Pre-loss growth: pre-loss version of the Chinese version of the Hogan Grief Reaction Checklist (C-HGRC)-personal growth subscale; Pre-loss grief: pre-loss version of the C-HGRC; Attachment avoidance: the Chinese version of the Experiences in Close Relationships–Relationship Structures questionnaire (ECR-RS)-avoidance subscale; Attachment anxiety: Chinese version of the ECR-RS-anxiety subscale; Relational closeness: the Inclusion of Other in the Self scale; Internalized CB: the Chinese Continuing Bond Scale-internalized subscale; Externalized CB: the Chinese Continuing Bond Scale- externalized subscale; Post-loss growth: HGRC personal growth subscale after loss; Post-loss grief: HGRC scores after loss.

Two hierarchical multiple regressions were conducted. Predicting variables significantly correlated with post-loss grief, namely pre-loss grief, externalized CBs, and attachment avoidance, were included in the first hierarchical multiple regression model. In step 1, the caregiver’s age, which was significantly correlated with post-loss grief, was entered. In step 2, pre-loss grief and attachment avoidance were entered. In step 3, externalized CBs were entered. The analyses revealed that the caregiver’s age had no significant impact on the level of post-loss grief [*R*^2^ = .06 (adjusted *R*^2^ = .04), *F* (1,63) = 3.66, *p* = .061]. The addition of pre-loss grief and attachment avoidance significantly improved the model of post-loss grief [*R*^2^ = .21 (adjusted *R*^2^ = .17), *F* (2,61) = 5.95, *p* = .004]. Pre-loss grief had a significant impact on post-loss grief, whereas attachment avoidance was negatively significantly associated with post-loss grief. The results of the third step of the analysis revealed that the addition of externalized CBs significantly improved the model of post-loss grief [*R*^2^ = .31 (adjusted *R*^2^ = .26), *F*(1,60) = 8.36, *p* = .005]. Age, pre-loss grief, and externalized CBs were significant predictions for a higher level of post-loss grief, whereas attachment avoidance was not. The result indicated that model 3 has provided the best explanation for the variance of post-loss grief. The results of hierarchical multiple regression analyses are summarized in Table 4.

**Table 4.** Hierarchical multiple regression analysis of post-loss grief (*N* = 66)

	Model 1			Model 2			Model 3	
Predictors	Δ*R*^2^	Standardized *β*		Δ*R*^2^	Standardized *β*		Δ*R*^2^	Standardized *β*
	.06			.15**			.10**	
Age		−.24			−.21			−.24*
Attachment avoidance					−.23*			−.19
Pre-loss grief					.32*			.27*
Externalized CB								.32**
Total *R*^2^(adjusted *R*^2^)	Model 3 = .31 (.26)							
*n*	66							

* *p* < 0.05.

** *p* < 0.01.

The second hierarchical multiple regression model included predicting variables significantly correlated with post-loss growth: pre-loss grief, pre-loss growth, and relational closeness. In step 1, the caregiver’s gender, which was significantly correlated with post-loss growth, was entered. In step 2, pre-loss grief and pre-loss growth were entered. In step 3, relational closeness was entered. The analyses revealed that the caregiver’s gender had no significant impact on the level of post-loss growth (*R*^2^ = .05 [adjusted *R*^2^ = .03], *F* [1,63] = 3.29, *p* = .075). The addition of pre-loss grief and pre-loss growth significantly improved the model of post-loss growth (*R*^2^ = .28 [adjusted *R*^2^ = .25], *F* (2,61) = 9.97, *p* < .001]. Pre-loss growth significantly impacted post-loss growth, whereas pre-loss grief did not (*p* = .094). The results of the third step of the analysis revealed that the addition of relational closeness did not significantly improve the model of post-loss growth [R^2^ = .29 (adjusted *R*^2^ = .24), *F*(1,60) = .35, *p* = .556], indicating that model 2 has provided the best explanation for the variance of post-loss growth. The results of hierarchical multiple regression analyses are summarized in Table 5.

**Table 5.** Hierarchical multiple regression analysis of post-loss growth (*N* = 66)

	Model 1			Model 2			Model 3	
Predictors	Δ*R*^2^	Standardized *β*		Δ*R*^2^	Standardized *β*		Δ*R*^2^	Standardized *β*
	.05			.23***			.00	
Gender(male)		−.24			−.19			−.20
Pre-loss grief					−.20			−.19
Pre-loss growth					.40***			.38**
Relational closeness								.07
Total *R*^2^(adjusted *R*^2^)	Model 2 = .29 (.25)							
*n*	66							

** *p* < 0.01.

*** *p* < 0.001.

## Discussion

The present study investigated the longitudinal effects of caregivers’ pre-loss grief, relational closeness, and attachment insecurities with the patients they cared for during the end-of-life care phase. We assessed caregivers’ pre-loss grief, pre-loss growth, attachment insecurities, and relational closeness during the palliative care phase. We also measured the caregiver’s post-loss grief, post-loss growth, and CB expressions 6–12 months after the patient’s death. The main findings are as folows: (1) Correlational analysis showed that caregivers’ level of attachment insecurities during the palliative care phase did not significantly correlate with CB expressions. Only attachment avoidance shows a negative correlation with post-loss grief, controlling for the caregiver’s age. (2) Hierarchical multiple regression analyses revealed that the caregiver’s age, externalized CBs, and pre-loss grief were significant predictors of post-loss grief. In contrast, attachment avoidance had no significant impact on post-loss grief. (3) For the hierarchical multiple regression model on post-loss growth, only pre-loss growth had a significant impact on post-loss growth.

First, attachment insecurities were not significantly associated with CB expressions and post-loss grief. Although attachment avoidance had a mild negative correlation with post-loss grief, there was no significant association between attachment avoidance and post-loss grief in the final regression model. The findings of this study are consistent with those of Eisma et al. (2023), which suggested that longitudinal analyses showed inconsistent evidence that insecure attachment styles lead to more grief symptoms. This calls for a reconsideration of the role of adult attachment in modern grief theories. The study also suggests that in a culture with deeply rooted Confucian values, conflicted family relationships (Kissane et al. 2006), as opposed to dyadic attachment relationships, may constitute stronger risk factors. We propose that future studies consider including the measurement of family relationships in the palliative care phase to examine the potential influence of family dynamics on the caregiver’s post-loss grief adjustment, thereby guiding future research and practice in this area.

Second, the results of our study support a significant relationship between externalized CBs and post-loss grief. Externalized CBs may serve as a strong predictor of post-loss grief because of the following 2 reasons: (1) Externalized CBs is an indicator of unresolved loss and complicated relationship with the deceased, as defined in the CBs theory and adult attachment literature (Stroebe et al. 2010). Because bereaved individuals may become dependent on their hallucinations and illusions of the deceased patient, externalized CBs can be viewed as a maladaptive coping strategy and a potential risk factor in the development of prolonged grief disorder in bereaved caregivers. (2) Externalized CBs may be a comforting behavior triggered by intense grief reactions. In such cases, grief is the cause of externalized CB expressions. It is worth noting that 2 new CB models have been proposed since 2020. One captures the overt behavior to continue bonds with the deceased person (Eisma & Nguyen, 2023), and another distinguishes symbolic and concrete CB to indicate normal and pathological grief, respectively (Sekowski 2021). More studies are needed to explore the relationship between these new CB concepts and grief adaptation.

Finally, our results support a significant relationship between pre- and post-loss growth. While relational closeness did not significantly impact post-loss growth in our final regression model, a previous study (Lee et al. 2023) found that the caregiver’s relational closeness with the patient during palliative care significantly predicts the caregiver’s pre-loss growth. A possible explanation might be that the close relationship between the caregiver and the patient results in greater pre-loss growth for the caregiver. This, in turn, could lead to increased post-loss growth for the caregiver after the patient has passed away. Nevertheless, more research evidence is needed to support this explanation. Future studies could adopt a longitudinal design to examine whether the caregiver’s pre-loss growth could mediate the relationship between relational closeness and post-loss growth.

### Study limitations

The first limitation of this study is its small sample size. This might be the reason why attachment insecurities were not significantly correlated with pre-loss and post-loss grief severity. Second, we did not gather data on the duration of patients’ hospice care and the specific timing of post-loss variable measurements. Third, the sample is dominated by university-educated females who have experienced the loss of a parent. This severely limits the generalizability of the results to other populations. Finally, we measured caregivers’ grief at only 1 follow-up time point. Future follow-up, longitudinal studies with grief assessments at multiple time points should be conducted to elucidate the mechanism of the transformation of caregivers’ relationships with the deceased patient as well as the potential mechanism underlying the association between CB expressions and grief adaptation.

### Clinical implications

Despite the limitations, this longitudinal study proves that the relationship quality between the caregiver and the patient is critical to caregivers’ post-loss adjustment. The strong predictability of externalized CB on the severity of post-loss grief also indicates a possible risk factor for prolonged grief disorder. Breen et al. (2020) revealed that it takes 9–10 months for caregivers to adapt to the impact of caregiving and bereavement, highlighting the need for palliative care services to support family caregivers in the caring phase and bereavement. Echoing with Breen et al. (2020), we suggest that psychosocial intervention routinely assesses the caregiver’s pre-loss grief and relationship quality with the patient. Besides, follow-up support might also be needed between 6 and 12 months after the death. An early intervention and follow-up psychosocial support for at-risk caregivers could help ameliorate the severity of post-loss grief and facilitate the bereavement adjustment.

## Conclusions

Caregivers’ pre-loss grief and continuing relationship with the patient/deceased are associated with their following post-loss adjustment. Our study demonstrated that the caregiver’s pre-loss grief and externalized CB expression could significantly predict the caregiver’s post-loss grief severity; pre-loss growth was significantly associated with the caregiver’s personal growth after the patient had passed away. Our findings have highlighted the importance of caregivers’ relational quality with the patient regarding caregivers’ bereavement adjustment.

## Data Availability

The data that support the findings of this study are openly available in OPENICPSR at 10.3886/E193181V1.

## References

[ref1] Aron A, Aron EN and Smollan D (1992) Inclusion of Other in the Self Scale and the structure of interpersonal closeness. *Journal of Personality and Social Psychology* 63(4), 596–612. doi:10.1037/0022-3514.63.4.596.

[ref2] Bowlby J (1969) *Attachment (Vol. 1)*. New York: Basic Books.

[ref3] Bowlby J (1973) *Attachment and Loss. Vol 2*. NewYork: Basic Books.

[ref4] Bowlby J (1980) *Attachment and Loss. Volume III: Loss: Sadness and Depression*. New York: Basic Books.

[ref5] Bowlby J (1982) *Attachment and Loss. Vol. 1: Attachment, 2nd Ed*. New York: Basic Books.

[ref6] Breen LJ, Aoun SM, O’Connor M, Johnson AR and Howting D (2020) Effect of caregiving at end of life on grief, quality of life and general health: a prospective, longitudinal, comparative study. *Palliative Medicine* 34(1), 145–154. doi:10.1177/0269216319880766. 31659934

[ref7] Cohen O and Katz M (2015) Grief and growth of bereaved siblings as related to attachment style and flexibility. *Death Studies* 39(3), 158–164. doi:10.1080/07481187.2014.923069. 25719968

[ref8] Eisma MC, Bernemann K, Aehlig L, et al. (2023) Adult attachment and prolonged grief: a systematic review and meta-analysis. *Personality and Individual Differences* 214, 112315. doi:10.1016/j.paid.2023.112315.

[ref9] Eisma MC and Nguyen LTH (2023) How we continue bonds with deceased persons: The proximity-seeking behavior scale. *Death Studies* 47(2), 164–171. doi:10.1080/07481187.2022.2039811 35188873

[ref10] Field NP and Filanosky C (2009) Continuing bonds, risk factors for complicated grief, and adjustment to bereavement. *Death Studies* 34(1), 1–29. doi:10.1080/07481180903372269. 24479173

[ref11] Field NP, Gao B and Paderna L (2005) Continuing bonds in bereavement: an attachment theory based perspective. *Death Studies* 29(4), 277–299. doi:10.1080/07481180590923689. 15849880

[ref12] Fraley RC, Heffernan ME, Vicary AM, et al. (2011) The experiences in close relationships-relationship structures questionnaire: a method for assessing attachment orientations across relationships. *Psychol Assessment* 23(3), 615–625. doi:10.1037/a0022898. 21443364

[ref13] Harrison O, Windmann S, Rosner R, et al. (2022) Inclusion of the other in the self as a potential risk factor for prolonged grief disorder: a comparison of patients with matched bereaved healthy controls. *Clinical Psychology and Psychotherapy* 29(3), 1101–1112. doi:10.1002/cpp.2697 34822735

[ref14] Ho SM, Chan IS, Ma EP, et al. (2013) Continuing bonds, attachment style, and adjustment in the conjugal bereavement among hong kong chinese. *Death Studies* 37(3), 248–268. doi:10.1080/07481187.2011.634086. 24524435

[ref15] Hogan NS and Schmidt LA (2015) Hogan Grief Reaction Checklist (HGRC). In *Techniques of Grief Therapy*. Taylor & Francis Group: Routledge, 39–45.

[ref16] Holm M, Årestedt K and Alvariza A (2019) Associations between predeath and postdeath grief in family caregivers in palliative home care. *Journal of Palliative Medicine* 22(12), 1530–1535. doi:10.1089/jpm.2019.0026. 31225778

[ref17] Ke (2021) interpersonal expansion scale: a single item measure of self-expansion. *Advance in Psychology* 11(02), 542–549. doi:10.12677/AP.2021.112061.

[ref18] Kim U, Yang G and Hwang KK (2006) *Indigenous and Cultural Psychology: Understanding People in Context*. New York, USA: Springer.

[ref19] Kissane DW, McKenzie M, Bloch S, et al. (2006) Family focused grief therapy: a randomized, controlled trial in palliative care and bereavement. *The American Journal of Psychiatry* 163(7), 1208–1218. doi:10.1176/ajp.2006.163.7.1208. 16816226

[ref20] Klass D, Silverman PR and Nickman SL (Eds.), (1996) *Continuing Bonds: New Understandings of Grief*. Philadelphia, PA, US: Taylor & Francis.

[ref21] Lee WL, Cheng CT, Hou YC et al, (2023) Pre-Loss Grief and Personal Growth in Taiwanese Cancer Patient Caregivers. *Journal of Loss and Trauma*, 29(3), 275–290. doi:10.1080/15325024.2023.2249402

[ref22] Lee WL, Yu HT and Lin YS (2024) Ethical bonds transformation in bereaved Taiwanese families: A preliminary study. *Omega*, 89(4), 1592–1608. doi:10.1177/00302228221097292 35465776

[ref23] Li Q and Loke AY (2013) The positive aspects of caregiving for cancer patients: a critical review of the literature and directions for future research. *Psycho‐oncology* 22(11), 2399–2407. doi:10.1002/pon.3311. 23712938

[ref24] Liljeroos M, Krevers B and Milberg A (2024) Family members’ long-term grief management: a prospective study of factors during ongoing palliative care and bereavement.*Palliative and Supportive Care* 22(5), 884–895. doi:10.1017/S1478951522001687. 36545770

[ref25] Lin KT (2016). *The performance of Rorschach inkblot test in adult attachment style: a verification of Fonagy’s psychodynamic theory* [Master’s thesis, National Chengchi University]. Taipei. https://hdl.handle.net/11296/9muzga (accessed 28 June 2025).

[ref26] Lindauer A and Harvath TA (2014) Pre‐death grief in the context of dementia caregiving: a concept analysis. *Journal of Advanced Nursing* 70(10), 2196–2207. doi:10.1111/jan.12411. 24702153

[ref27] Nicholls W, Hulbert-Williams N and Bramwell R (2014) The role of relationship attachment in psychological adjustment to cancer in patients and caregivers: a systematic review of the literature. *Psycho-Oncology* 23(10), 1083–1095. doi:10.1002/pon.3664. 25156192

[ref28] Nielsen MK, Neergaard MA, Jensen AB, et al. (2017) Predictors of complicated grief and depression in bereaved caregivers: a nationwide prospective cohort study. *Journal of Pain and Symptom Management.* 53(3), 540–550. doi:10.1016/j.jpainsymman.2016.09.013. 28042073

[ref29] Palacio C and Limonero JT (2020) The relationship between the positive aspects of caring and the personal growth of caregivers of patients with advanced oncological illness. *Supportive Care in Cancer* 28, 3007–3013. doi:10.1007/s00520-019-05139-8. 31823055

[ref30] Prigerson HG, Horowitz MJ, Jacobs SC, et al. (2009) Prolonged grief disorder: psychometric validation of criteria proposed for DSM-V and ICD-11. *PLoS Medicine* 6(8), e1000121. doi:10.1371/journal.pmed.1000121. 19652695 PMC2711304

[ref31] Root BL and Exline JJ (2014) The role of continuing bonds in coping with grief: overview and future directions. *Death Studies* 38(1), 1–8. doi:10.1080/07481187.2012.712608. 24521040

[ref32] Sekowski M (2021) Concrete and symbolic continuing bonds with a deceased person: the psychometric properties of the continuing bonds scale in bereaved surviving family members. *Journal of Social & Personal Relationships* 38(5), 1655–1670. doi:10.1177/02654075211001574.

[ref33] Sękowski M, Ludwikowska-Świeboda K and Prigerson HG (2024) Prolonged Grief Disorder in ICD-11 and DSM-5-TR: factor structure, and psychosocial and loss-related correlates in a sample of widowed persons. *Psychiatria Polska* 58(2), 265–276. doi:10.12740/PP/159024. 39003510

[ref34] Sekowski M and Prigerson HG (2022) Disorganized attachment and prolonged grief. *Journal of Clinical Psychology* 78, 1806–1823. doi:10.1002/jclp.23325. 35132649

[ref35] Sekowski M and Prigerson HG (2022b) Conflicted or close: which relationships to the deceased are associated with loss-related psychopathology? *British Journal of Clinical Psychology* 61(2), 510–526. doi:10.1111/bjc.12344. 34724233

[ref36] Singer J, Roberts KE, McLean E, et al. (2022) An examination and proposed definitions of family members’ grief prior to the death of individuals with a life-limiting illness: a systematic review. *Palliative Medicine* 36(4), 581–608. doi:10.1177/02692163221074540. 35196915 PMC10098140

[ref37] Stroebe M, Schut H and Boerner K (2010) Continuing bonds in adaptation to bereavement: toward theoretical integration. *Clinical Psychology Review.* 30(2), 259–268. doi:10.1016/j.cpr.2009.11.007. 20034720

[ref38] Tang Y (2019) Challenges, personal growth and social support among family caregivers of terminally ill cancer patients in Southern China. *Qualitative Social Work* 18(4), 638–654. doi:10.1177/1473325018755890.

[ref39] Treml J, Schmidt V, Nagl M, et al. (2021) Pre-loss grief and preparedness for death among caregivers of terminally ill cancer patients: a systematic review. *Social Science & Medicine* 284, 114240. doi:10.1016/j.socscimed.2021.114240. 34303292

[ref40] Tsilika E, Parpa E, Zygogianni A, et al. (2015) Caregivers’ attachment patterns and their interactions with cancer patients’ patterns. *Supportive Care in Cancer* 23(1), 87–94. doi:10.1007/s00520-014-2329-6. 24989321

[ref41] Wayment HA and Vierthaler J (2002) Attachment style and bereavement reactions. *Journal of Loss &trauma* 7(2), 129–149.

[ref42] Yu W, He L, Xu W, et al. (2016) How do attachment dimensions affect bereavement adjustment? A mediation model of continuing bonds. *Psychiatry Research* 238, 93–99. doi:10.1016/j.psychres.2016.02.030. 27086217

[ref43] Zordan RD, Bell ML, Price M, et al. (2019) Long-term prevalence and predictors of prolonged grief disorder amongst bereaved cancer caregivers: a cohort study. *Palliative and Supportive Care* 17(5), 507–514. doi:10.1017/S1478951518001013. 30767818

